# Seeing Is Believing: Making Wellbeing More Tangible

**DOI:** 10.3389/fpsyg.2022.809108

**Published:** 2022-03-14

**Authors:** Dianne A. Vella-Brodrick, Anneliese Gill, Kent Patrick

**Affiliations:** Centre for Wellbeing Science, Melbourne Graduate School of Education, University of Melbourne, Parkville, VIC, Australia

**Keywords:** positive psychology, positive education, wellbeing science, interventions, biofeedback, physiology, interdisciplinary

## Abstract

Positive Psychology has been instrumental in promoting wellbeing science in the modern era. However, there are still ways in which positive psychology interventions and positive education programmes can be improved to achieve more robust and sustained effects. One suggested method is to make wellbeing more salient and tangible through the use of objective tools that assess the relationship between psychological and physiological wellbeing, and enable wellbeing status and change to be seen. With the addition of an interdisciplinary team, as well as technology-enabled and pedagogically sound learning tools and approaches, the potential for positive outcomes and impact increases exponentially. Monitoring wellbeing progress in this way can provide evidence, motivation and belief in positive psychology and wellbeing interventions. This can lead to engaged learning, sustained benefits and systemic impact. Positive psychology needs to strategically extend on the emerging work in this field to help everyone, including policy makers, notice and value wellbeing.

## Introduction

Positive psychology has made many important contributions to wellbeing science, including spotlighting research focused on understanding ‘what makes life worth living’ and examining how to ‘thrive, not just survive’ (Seligman and Csikszentmihalyi, 2000). These catch phrases are now well cited in wellbeing literature. At the very least, founding scholars and champions of positive psychology have raised academic and public awareness about the biased attention on psychopathology and mental illness within psychology (Seligman and Csikszentmihalyi, 2000; Westerhof and Keyes, 2010) and the simultaneous need to focus on the presence of wellbeing (flourishing) for people to experience good mental health (Keyes, 2002).

Although some of the core principles underlying positive psychology, such as focusing on emotion regulation, strengths and fostering eudaimonic wellbeing, may not be novel (Vella-Brodrick, 2013, 2016), there are areas in which positive psychology has made unique contributions beyond bringing more attention to the positive side of the mental health spectrum (Seligman, 2002). However, it will be argued that after two decades since its inception, positive psychology needs to innovate. Interdisciplinary, objective and technology-supported approaches are suggested to expand and complement what already exists. In this paper, we will first briefly explore the unique contributions of positive psychology; then, we will identify key issues and areas where more progress is needed and finally, we will conclude with some examples of work already commenced in these recommended areas.

## Unique Contributions of Positive Psychology

Two noteworthy areas where positive psychology has advanced wellbeing science include the development of wellbeing measures and the proliferation of positive psychology interventions (PPIs), including positive education programmes (Vella-Brodrick, 2013; Slemp et al., 2017; Vella-Brodrick et al., 2020). With regards to measures, the development of standardised scales is evident in a number of key areas of positive psychology including strengths (Peterson and Seligman, 2004), meaning (Steger et al., 2006), gratitude (McCullough et al., 2002), flourishing (Diener et al., 2009) and hedonic and eudaimonic wellbeing (Delle Fave et al., 2011). The development of these standardised scales has enabled considerable progress to be made with measuring wellbeing, identifying correlates of wellbeing and evaluating the effectiveness of wellbeing interventions (Vella-Brodrick, 2013).

A concurrent undertaking has involved the development of a range of PPIs and positive education programmes focused on expressing gratitude, identifying and using character strengths, meaning making and building hope, such as imagining your ‘best possible self’ (Seligman et al., 2005; Slemp et al., 2017). Studies examining the efficacy of these interventions and programmes have found favourable results in a diverse range of wellbeing outcomes including happiness, satisfaction with life, social and emotional skills, competence and autonomy (McCullough et al., 2002; Seligman et al., 2005; Vella-Brodrick et al., 2021). Meta-analyses also support these subjective and psychological wellbeing benefits (Sin and Lyubomirsky, 2009; Boiler et al., 2013; Carr et al., 2020; Tejada-Gallardo et al., 2020); however, the effect sizes have been relatively small to moderate (White et al., 2019). Although strong effects are rare in wellbeing or prevention science interventions, and context-relevant comparisons are encouraged (Tanner-Smith et al., 2018), these findings of around 0.20 do not meet the average effect size (or hinge point) for school-based learning interventions, which according to Hattie’s (2011) work is 0.40. It is incumbent on any scientific field that examines a fundamental human quality such as wellbeing, to optimise the intervention effects for as many people, in as many contexts as possible. Positive psychology is now at the stage where it needs to strategically build on its foundational work by diversifying and advancing its methods and interventions.

## Time for More Progress and Key Issues

Recently, there have been calls for positive psychology to adopt a more interdisciplinary and systemic approach to studying wellbeing (Kern et al., 2020; Mead et al., 2021). Developing a broader conceptualization of wellbeing that moves beyond the positive psychology lens by integrating complementary disciplines and theories could lead to improved understanding and innovative approaches to enhancing wellbeing. Collaborating with others from different disciplines or approaches who also study and value wellbeing, and to identify best practices that could be adopted or combined to optimise the positive effects of wellbeing strategies seems a logical next step. Disciplines and fields of inquiry such as sports science, neuroscience, medicine, psychology, education, policy, technology, positive youth development and social and emotional learning may have developed theory, methods, measures and interventions that can complement or integrate with positive psychology practices to yield more powerful benefits or engaging methods. Lyubomirsky (2008) identifies the importance of person-activity fit and variety to maintain interest and benefits. There are some examples of this integration starting to emerge. For example, Nisbet et al. (2019) combined a nature intervention with mindfulness. She found better wellbeing outcomes for the combined intervention relative to the same nature intervention without mindfulness. Peters et al. (2010) combined mental imagery with the ‘best possible self’ writing intervention and found significant effects. These examples illustrate how strategies from various disciplines can be combined to achieve improved outcomes or increased variety for sustained benefits. However, many scholars working within positive psychology tend to remain within their disciplinary ‘boundaries’ even though the topic of wellbeing lends itself nicely to interdisciplinary work.

Another issue with wellbeing interventions including PPIs is the high level of engagement and training required to facilitate genuine learning and enable skill transfer when needed. It is important that PPIs ‘stick’ so that they can help create significant and sustained improvements to the individual’s life. A clear pedagogical learning framework and the latest technological innovations can improve the learning process and make skill development through training more enticing. For example, a meta-analysis of social and emotional learning interventions in school settings found that programmes adopting a sequenced, active, focused and explicit (SAFE) framework for learning were more effective than programmes not following this process (Durlak et al., 2011). Programmes that have clear learning intentions and clear feedback about progress are also deemed to be of better quality. For example, Hattie (2011) stresses the importance of making the learning experience visible to both teachers and students and to help students to become their own teachers over time so that they can be passionate about learning and make it a lifelong pursuit. Seeking and acting upon feedback are an important theme associated with effective learning.

Increasingly, technological advances are being used to enhance learning experiences. The onset of the Internet and the rapid development of portable, affordable and scalable devices have seen Technology-Enabled Learning (TEL) become commonplace in educational settings (Kirkwood and Price, 2016). Despite this, using technology alone as a novel replacement for traditional learning frameworks will not necessarily be successful in the long-term unless combined with a solid pedagogical rationale (Ferguson, 2019). Ferguson (2019) points out that without this solid rationale, the novelty of using new technological approaches will fade over time and positive change will not be sustained. However, the use of innovative technological approaches to support sound pedagogical practice can result in positive outcomes. An example of a technological innovation that has the potential to enhance traditional pedagogical practices is using video games to enhance learning. Gamification can provide incidental learning which is augmented by providing challenges and rewards in an engaging and immersive environment (Sharples, 2019) thus making it more likely that users will be kept motivated and engaged in the learning process (Weerdmeester et al., 2020). The use of technology can heighten interest, enjoyment and learning efficiency (Liang and Xiaoming, 2013; Lee et al., 2017). Serious games with intentional learning principles can increase motivation to learn and improve performance (Gee, 2009; Laamarti et al., 2014). For example, Chen and Hsu (2019) exposed a group of 66 college students to a serious game titled *Slave Trade* to examine if vocabulary and history knowledge improved. They found significant improvements in both areas from pre- to post-game and also found that participants reported a positive learning experience through the gaming environment.

In addition, technological advances have allowed us to access a range of information about how we respond to the world around us. Ubiquitous ‘smart’ devices or ‘wearables’ collect an ongoing stream of health and wellness information (e.g., activity, sleep and heart rate) to enable us to monitor and track our health status (Weerdmeester et al., 2020). This information can help combat the abstract nature of subjective wellbeing where it can be difficult to quantify and see change, and thus motivate individuals to enhance their wellbeing. The need for objective data is becoming inescapable. Indeed, we are in the era of what is being described as the ‘quantified self’ where everything about a person and their responses to their environment is being measured (Lupton, 2016).

Physiological data that are collected from smart devices can also be used as a form of biofeedback, where individuals can monitor their personal data and learn how to adapt physiological functioning to improve other aspects of their health (e.g., mental health). Subsequently, biofeedback is increasingly being incorporated in smartphone self-monitoring apps (Weerdmeester et al., 2020; Leonidis et al., 2021) which use the health and wellness information to provide personalised nudges to help individuals adapt. Given the breadth of the data that is currently being collected by wearables it also provides an opportunity for a more holistic approach to biofeedback in which several health and wellness markers could be used to provide accurate personalised guidance. This approach has been used in the design of Intelligent Homes that use real-time data collected by wearables, as well as information received from various sensors in the house, to provide personalised relaxation and sleep advice (Leonidis et al., 2021). The system ‘…aims to detect, as unobtrusively as possible, whenever a user is stressed and try to help him/her relax by exploiting a variety of devices…’ (Leonidis et al., 2021, p. 12). This holistic approach, which incorporates multiple health and wellness markers (e.g., sleep, physical activity, nutrition and sunlight), as well as other more objective measures, may provide a direction for the development of future biofeedback apps.

To date, the science of positive psychology and wellbeing has remained largely in the ‘subjective’ and introspective realm. Hence, individuals may lack belief in and motivation to engage with wellbeing strategies because the processes and benefits are not immediately visible to them. Positive psychology needs to consider these consumer behaviours around the quantified self, best practice education (pedagogy) and technology-enabled learning to consider ways of making learning about wellbeing more tangible and salient so that it is valued. While the more nebulous subjective experience is a significant part of psychological wellbeing and should not be abandoned simply because it is complex to measure and understand, there are ways of integrating objective wellbeing data to add another dimension to the wellbeing experience and the way it is understood and translated into practice. The mind–body connection seems to be a good starting place.

Monitoring our physiology can provide relatively accurate insights—not only about our physical condition but also our psychological health and wellbeing given that physiology and psychology are inextricably linked (Niedenthal, 2007). This aligns with the perspective that the mind and body are connected. The way we think, behave and function physiologically are all interconnected (Weerdmeester et al., 2020). For example, how we identify and interpret physiological indices has an effect on how we respond emotionally (Gross, 2002; Kanbara and Fukunaga, 2016). It has been suggested that when these systems are coherent and perceived stress matches the concurrent physiological response, it can lead to better adaptive functioning (see Sommerfeldt et al., 2020). For instance, individuals who display greater coherence of subjective stress and electrocardiogram recorded heart rate have been found to exhibit higher levels of psychological wellbeing (Sommerfeldt et al., 2020). Furthermore, low stress-heart rate coherence can be an indicator of maladaptive coping strategies such as denial (Sommerfeldt et al., 2020).

The connection we have with our inner selves, including physiological processes, is known as interoceptive awareness. Those who report low levels of interoceptive awareness (e.g., introspection) are less likely to be self-connected; that is, they are less likely to be self-aware, have self-acceptance and align their behaviour with their true self (Klussman et al., 2020). Two types of barriers to self-knowledge have been identified, informational and motivational. Informational barriers refer to limited or inaccurate details about the self, whereas motivational barriers refer to perceptual biases such as self-enhancement and self-verification of pre-existing beliefs (Vazire, 2010). In addition to these biases, individuals report that they do not have time or space in their busy schedules to become self-connected. According to Klussman et al. (2020) objective self-observation and knowledge can enhance self-connection.

Advances in technology and neurophysiological devices make it possible to measure internal bodily states with the intent of gaining awareness and taking control over processes previously outside the individual’s level of awareness. Known as biofeedback this objective, real-time information can make wellbeing more tangible and appealing, increasing motivation and engagement. Thus, incorporating this additional pathway to self-awareness has the potential to significantly impact the way we approach mental health and wellbeing promotion.

## Examples of Tangible Wellbeing in Progress

There has been some emerging work examining how the mind and body are connected among positive psychology and medical scholars. For example, Kok et al. (2013) identified how emotional health can influence physical health (vagal tone). Similarly, studies examining heart rate variability (HRV) and the role of the vagus nerve in relation to psychological wellbeing have been undertaken. The vagus nerve is the largest autonomic nerve of the central nervous system and has a direct role in parasympathetic control, regulating heart rate, respiration and digestion. Higher vagal activity is associated with greater autonomic flexibility or the capacity of the autonomic nervous system to change according to environmental needs (Friedman and Thayer, 1998; Porges, 2007). Heart rate variability is often used as an indirect measure of vagal tone or activity (Marmerstein et al., 2021). Higher vagal tone has been linked with improved emotional regulation (Thayer et al., 2009), pro-social behaviour (Eisenberg et al., 1995), self-regulation (Park and Thayer, 2014) and adaptive coping resources (Porges et al., 1994). In addition, it has been proposed that there is a reciprocal relationship between wellbeing and vagal activity, whereby positive social and emotional experiences support autonomic health and vice versa (Kok and Fredrickson, 2010; Kok et al., 2013). Thus, HRV can provide people with information about their autonomic functioning and wellbeing.

Adopting a more extensive systems approach, Mead et al. (2021) have proposed a transdisciplinary model of wellbeing science that encompasses individual, community and environmental domains and physical and socio-contextual factors that may positively or negatively impact peoples’ experiences and cultivation of wellbeing. They highlight the importance of self-connection which can be enhanced by developing greater awareness or understanding of the self (Klussman et al., 2020; Mead et al., 2021). Although traditionally this might refer to an understanding of beliefs, values, goals and behaviour, it could also be extended to incorporate physical and physiological factors. Central to supporting this self-connection is the vagal nerve as it provides a structural link between the mind and body (Mead et al., 2021). What this work suggests is the need to progress towards assessing coherence levels among different measures that contribute to a more holistic understanding of wellbeing. Integrating psychological states with physiological response data can provide more complete information than is possible with a single measurement approach (Sommerfeldt et al., 2020).

Similarly, Davidson’s (2021) work at the Centre for Healthy minds underscores that individuals’ perceptions and feelings of stress can differ from their physiological response to stress. Their work has found that individuals whose subjective experience of stress aligns strongly with physiological changes in their body (i.e., have strong stress-heart rate coherence) tend to have higher psychological and physical wellbeing. They are now exploring whether interoceptive accuracy of heart rate is a prerequisite for physiological and subjective experience coherence, as well as whether mindfulness practice can enhance this coherence. In response to their comment that the field of psychological wellbeing ‘lacks a unifying framework that clarifies the dimensions of human flourishing that can be cultivated’ Dahl et al. (2020, p. 32197) have proposed a four pillar framework focused on the plasticity of wellbeing that includes awareness, connection, insight and purpose. In so doing, they include evidence from clinical psychology, wellbeing science and neuroscience, and encourage future wellbeing researchers to move beyond insular and self-report approaches to consider how psychological and biological mechanisms as well as new technology might increase our understanding and promotion of human flourishing.

We at the Centre for Wellbeing Science are focusing on the mind–body connection as a way to help individuals engage more fully with wellbeing education. For example, our Bio-Dash wellbeing programme integrates biofeedback first as a monitoring tool to enhance interoceptive awareness and identify areas of strength and improvement, and secondly to enhance the wellbeing learning process by gaining feedback about how the application of the learning is progressing, not just subjectively but also at a neurophysiological level that can be observed and controlled. Bio-Dash integrates the learnings from a variety of models that are aimed at increasing motivation and engagement to participate in wellbeing interventions. These include the promotion of person-activity fit (Lyubomirsky, 2008; Lyubomirsky and Layous, 2013), self-concordant motivation and need striving (Sheldon and Elliot, 1998, 1999), self-determination (Ryan and Deci, 2000), growth mindset (Dweck, 2008) and visible learning (Hattie, 2011). These approaches collectively emphasise the need for variety, choice, intrinsic motivation, self-efficacy, being open to learning and skill development, and providing clear, personalised feedback. In addition, Bio-Dash includes a range of virtual and real challenges to help transfer the learning in applied settings and scenarios, as well as gamification features that provide metrics such as time taken to complete a task, time spent in stress, neutral and relaxed states and rewards when relaxation goals are achieved. As found by Chen and Hsu (2019) adopting these tangible and creative learning strategies can enhance learning engagement so that it is a more positive experience. This can promote more practice, knowledge and skill development and overall wellbeing habits that can be applied in practical situations, as reported by nearly 200 students from a private boy’s school in Melbourne, who trialled and provided anonymous feedback on the Bio-Dash. Many identified the technological aspects of Bio-Dash (biofeedback and gamification) as novel features that helped to keep them motivated to learn more about the wellbeing strategies (see West et al., 2021 for further details about the Bio-Dash).

Aside from connecting the mind and body, there are other ways of making wellbeing more tangible, especially if an interdisciplinary perspective is adopted. For example, Stanley et al. (2021) have monetized wellbeing using a sustainability analysis framework including the economic, social and environmental trade-offs to support improved transport policy. This work has extended the cost–benefit analysis beyond traditional economic measures to show in dollar terms, the significant value of wellbeing to socio-economic outcomes. It also highlights the strength of interdisciplinary collaborations, whereby scholars with expertise in economy, social work, transport policy and psychology have infused their approaches to create new and valuable knowledge in a language that will appeal to different but highly relevant audiences. This reinforces a systems approach which speaks to policy makers and government and is critical for sustained benefits.

Along these lines, we recognise that wellbeing is not housed exclusively in an individual but in a complex system (Kern et al., 2020). Hence, building a culture of wellbeing, where it is readily expressed can make wellbeing more salient and part of everyday life. There is a growing understanding that people, groups and communities can express their level of wellbeing in a variety of ways, including through creative outlets such as writing and artistic works (e.g., singing, song writing, painting and poetry) and that this type of wellbeing expression can be learned (Oades and Johnston, 2017; Oades et al., 2020). These varied forms of expression become physical representations of wellbeing that can be seen, experienced and measured in terms of their ability to provoke different emotions, cognitions and behaviours that relate to wellbeing. Quantifying the extent to which people communicate, comprehend and compose wellbeing—wellbeing literacy—can provide a set of metrics that people can see and use as a point of comparison in the future, particularly in response to wellbeing interventions (see Oades et al., 2020). This can also enable cultural diversity to be more readily identified and understood.

## Conclusion

When individuals can witness firsthand how intentional effort to enhance wellbeing, through evidence-based approaches, actually influences the ‘hardwiring’ and neurophysiology of the body and experience the benefits of mind–body coherence, the value of wellbeing increases. When dollar values are placed on wellbeing such that the benefits of wellbeing initiatives outweigh the costs, this also attracts attention from policy makers and government. When we start to see and measure expressions of wellbeing in daily life and the ways in which these align with, for example, pro-social and civic behaviours across diverse cultures, this can have socio-political value. There is growing empirical evidence revolving around psychosocial and neurophysiological factors to support the sustained benefits of wellbeing interventions Rickard and Vella-Brodrick (2014). Cumulatively, these interdisciplinary, creative and objective approaches enable a case to be built for why wellbeing is important beyond the subjective, individual experience, and demonstrate that these outcomes are not imagined but are very real. Positive psychology has an important role to play in bringing together interdisciplinary work that draws on subjective and objective wellbeing methods and the latest technology. This will make wellbeing more tangible and help individuals to see and believe that they can have access to data and skills to improve their wellbeing.

## Data Availability Statement

The original contributions presented in the study are included in the article/supplementary material, further inquiries can be directed to the corresponding author.

## Author Contributions

DV-B conceived of the paper idea, designed the paper outline and content, prepared a first draft of the paper, made subsequent edits to the final stage, and submitted the paper. AG contributed to the writing and edits and assisted with the paper formatting. KP contributed to the writing and edits as well as proofreading. All authors contributed to the article and approved the submitted version.

## Conflict of Interest

The authors declare that the research was conducted in the absence of any commercial or financial relationships that could be construed as a potential conflict of interest.

## Publisher’s Note

All claims expressed in this article are solely those of the authors and do not necessarily represent those of their affiliated organizations, or those of the publisher, the editors and the reviewers. Any product that may be evaluated in this article, or claim that may be made by its manufacturer, is not guaranteed or endorsed by the publisher.
